# 

**Published:** 1891-07-11

**Authors:** 


### NOTICE TO CORRESPONDENTS.

All MS., Letters, Books for Review, and other matters intended for the Editor should be addressed The Editor, The Lodge, Porchester Square, London, W.

The Editor cannot undertake to return rejected M.S., even when accompanied by stamped directed envelope.

**Notice to Advertisers and Subscribers.**

All Advertisements, Orders for Copies of The Hospital, and Business Communications should be addressed to The Publisher, *not to the Editor*, The Hospital, 140, Strand, W.C.

The Cost of Subscription, post free, is as follows:—Per week, 1½d.; per quarter, 1s. 8d.; per half-year, 3s. 3d.; per annum, 6s. 6d. Foreign:—Per annum, 8s. 8d.; payable, in all cases, in advance.

**NOTICE.—The Index for Vol. IX.. (ending March, 1891) is on sale at the Office of this Journal, price 2½d., post free.**

### General Charities.

[This Column is devoted to an account of work of charitable institutions other than Medical Charities. The Editor will be glad to receive reports or news of work at 140, Strand. *Philanthropy* should be plainly written on the envelope.]

The Earl of Aberdeen presided over the forty-ninth annual meeting of **THE FIELD LANE RAGGED SCHOOLS**, which at present numbers fifty-four distinct sections of work, while upwards of 6,200 persons are benefited annually. The attendances at all the various classes, services, meetings, &c., show considerable advance, but in spite of much care the expenditure has been larger than the income. The servants' training home is perhaps one of the most useful of the society's undertakings, as is also the maintenance and training of the girls and boys in the industrial home.

Among the institutions which should be supported by those for whom an appeal is annually made to the public is **THE CABDRIVERS' BENEVOLENT ASSOCIATION**, but it does not appear to receive from cabdrivers the support it should, for the widow and orphan fund, which has now been in existence for eleven years, has, according to a statement made by the Secretary at the recent festival of the society, received up to the present very little sympathy or help from those most interested. The Committee make an earnest appeal to cabmen to give the fund their hearty support, but in justice to these men it should be remembered that competition is now-a-days very keen and that it is no easy matter for a cabman to make more than a living out of his trade, even if he is fortunate enough to be able to do that. It appears that out of the large number of cabdrivers in London, only 1,200 have become members. As a payment of five shillings a-year constitutes membership, it is clear that there must be many more who can afford, if they have the will, to contribute from their earnings. The public are always generously disposed, and rightly so, to those who are ready to help themselves. During the past year 89 persons were assisted with gifts, and 73 with loans, and annuities to the extent of £976 were granted to aged and infirm members of the fund. No one will deny the excellent character of the society, more especially as the proportion of cost in working the institution is very small in comparison with the work done.

The sunshine of the past month must have increased the expectant longing of many London children for a few hours or days in the beautiful country, of which so many have heard, but which so few have been fortunate enough to see. It is always a pleasant sight to see the bright little faces in the brakes which carry the children to their Eldorado, but why in the name of all that is comfortable should children who are being sent into the country for a day's fresh air be packed densely in a brake, which on a bright and hot day is shrouded in a heavy waterproof tarpaulin? A few days ago a party of children was to be seen passing through Trafalgar Square; in one of the vehicles was a heavy waterproof flapping on the side in the children's faces, taking away half their pleasure and all their comfort, and creating a stifling atmosphere, the effect of which was only too visible on the little ones' faces. The exercise of a very little common sense on the part of those responsible for the management of the excursion would have led them to have the waterproof fastened on the roof of the brake ready to be let down if necessary. Half a loaf is no doubt better than no bread, but we wish that funds were forthcoming to enable more children to be sent into the country for a fortnight. Now is the time for those who wish and are well enough off to contribute to the happiness of the poor children of the metropolis to send their contributions. Sir John Lubbock and Lord Kilmarnock, trustees to **THE CHILDREN'S COUNTRY HOLIDAYS FUND**, appeal urgently for help. They write that "Last year the Children's Country Holidays Fund sent 23,771 children into country cottages for not less than a fortnight's fresh air. This season, after the long, cold winter, many more children will need some country sun. Several new local committees have been formed in parts of London to which it has previously been impossible to extend the fund, and more help than ever will be needed this year, both in money, and in personal work. Local visitors find those children most needing to escape from London bricks and mortar, and arrange for a motherly welcome for them in some of the 600 villages where friendly country residents have found cottagers able and willing to give them a share of their country life. The parents contribute according to their means, and the fund does not pauperize the poor, while the enjoyment which the holiday gives to the children is incalculable. Our new census returns show a decrease of rural, and enormous increase of town, population, but most people will hardly realise the full meaning of this change. The town life is faster, more attractive, but undiluted it will not rear the same standard of Englishmen as in the past. The children are growing up in the slums in an ignorance quite inconceivable of the world of nature, and the slight amount of book learning which the schools endeavour to inculcate is a poor exchange for acquaintance with the world of fact. This year the fund is some hundreds of pounds behind the amount subscribed at the same season last year. A fortnight's holiday can be given for 10s. Will not some of our readers give that change to London children by sending donations to the Hon. Alfred Lyttelton, at 10, Buckingham Street, Strand, W.C.?"

### Scraps and Gleanings.

The Metropolitan Hospital Sunday collections now exceed £41,000.

At a meeting at Wirksworth £62 was raised for the new Derby Infirmary.

Four executions by electricity have taken place in Sing-Sing prison this week.

A very successful Hospital Saturday Parade has been held at High Wycombe.

The working men of Grimsby gave £131 to the hospital during the last twelve months.

A telegram from Alexandria states that typhus fever has broken out among the Mecca pilgrims.

The Duchess of Albany visited the British Home for Incurables last week, and went all over the building.

There are 40 cases of sunstroke in the military hospital at Erfurt owing to a forced march on a hot day.

Clackmanan County Hospital reports an income of £353, and an expenditure of £369 for the last twelve months.

Mr. John Furley was given a public dinner on Tuesday, in recognition of the aid he has rendered to ambulance work.

C. C. Whitefoord, surgeon, has been sentenced to five years' penal servitude for conspiring to perform an illegal operation.

Barnsley Beckett Hospital has a steady increase of patients yearly; luckily the Hospital Saturday and Sunday Funds increase also.

Great Yarmouth Hospital treated last year 379 in-patients and 3,500 out-patients. An eye department has lately been added to the hospital.

Dr. Charles Edward Sheppard committed suicide on June 30th, during a period of depression brought on by the death of one of his patients under chloroform.

The Church Extension Association, amongst its other good works, has a large convalescent home for children at Broadstairs, which during this season holds 300 inmates.

Enfield Cottage Hospital issues a glowing report; it is apparently quite satisfied with itself, and the people of the neighbourhood seem equally satisfied and subscribe liberally.

The Greenwich Hospital pension of £50 a-year for Fleet or Staff Surgeons rendered available by the death of Deputy-Inspector Fuller has been conferred on Deputy-Inspector-General John T. Caddy.

The death is announced, in her one hundred and third year, of Mrs. Boyack, a lady of considerable means, who had resided for many years at Bridge, near Canterbury. She was born in September, 1788.

The General Practitioners' Union held an interesting meeting last Tuesday to consider the questions of Hospital Management and the representation of General Practitioners on the General Medical Council. We hope to give particulars next week.

Darlington Fever Hospital is erecting a new disinfecting chamber at a cost of £400. During last autumn there was a typhoid epidemic, and the resources of the hospital were terribly taxed, and a shed had to be erected in the grounds. Dr. Barry says the epidemic was due to the water supply.

Messrs. Agnew and Sons have kindly lent their large galleries in Bond Street for the ladies' reception rooms on the occasion of the Congress on Hygiene and Demography to be held in London in August. Members of the Ladies' Committee will be present every day to receive the foreign and other visitors.

The question of an Infectious Diseases Hospital has been under discussion now for several years in Dartmouth, at first by the Sanitary Committee of the Town Council, and latterly by the new Port Sanitary Authority for Totnes and Dartmouth district. Difficulties have continually been cropping up as to a shore site, and now the authority has been compelled to fall back on a floating hospital as the only remaining course open.

Hospital Sunday was fully observed at Norwich. The Mayor, Sheriff, and Corporation went in state to the morning service at the Cathedral, and were met by the Dean and Canons, with the choristers and lay clerks, at the Grammar School, the choir then heading the procession into the Cathedral, the National Anthem being sung as a processional. In the evening the Bishop of Norwich preached on behalf of the same Fund.

The Metropolitan Asylum and London Fever Hospitals contained 866 scarlet-fever patients at the end of last week, against 872 and 891 on the preceding two Saturdays; 91 cases were admitted during the week, against 84 and 76 in the preceding two weeks. The deaths primarily attributed to influenza, which had declined from 319 to 117 in the preceding six weeks, further fell last week to 56.

At a meeting of the Lisburn, co. Antrim, Board of Guardians it was reported that a man named Evans had been for some time past suffering from a disease which is, beyond doubt, leprosy. It is supposed the man became infected in India, where he spent many years. It is feared that one of his relatives has caught the disease. The Local Government Board has given orders for the isolation of the unfortunate man.

Lord Carrington distributed the prizes to the successful students at Charing Cross last week. Mr. Stanley Dean read the report, which stated that last year they had expected to have had their new premises complete, but they were bitterly disappointed. The Hospital Association had acted very liberally to them. The year had been very active and satisfactory, as was shown by the fact that the average daily attendance was 220.

Dr. Lannelong made an important communication to the Academy of Sciences relative to a new method of treatment for tuberculosis. It consisted, he explained, in the application of chloride of zinc, which produced a transformation of morbid tissue and the almost immediate formation of numerous cellular tissues. Dr. Lannelong has applied his remedy to twenty-three persons affected in the articulations of the joints, and all these are now cured.

### THE STUDENT OF MEDICINE.

[All communications for this Supplement should be addressed to THE EDITOR of THE HOSPITAL, at 140, Strand, with the words, "Students' Column," written in the left hand top corner of the envelope.]

### EDITORIAL NOTES.

THE INSANITY OF GENIUS.—Professor Huxley, commenting upon the new book on "The Insanity of Genius," by Mr. Nisbet, writes: Genius, to my mind, means innate capacity of any kind above the average mental level. From a biological point of view, I should say that a genius among men stands in the same position as a "sport" among animals and plants, and is a product of that variability which is the postulate of selection, both natural and artificial. On the general ground that a strong, and therefore markedly abnormal, variety is *ipso facto* not likely to be so well in harmony with existing conditions as the normal standard (which has been brought to what it is largely by the operation of those conditions), I should say that a large proportion of "genuine sports" are likely to come to grief physically and socially, and that the intensity of feeling, which is one of the conditions of genius, is especially liable to run into insanity.

SWALLOWING A THERMOMETER.—This is the heading of a recent telegram from New York. According to the story a certain patient was in charge of a trained nurse in some part of the continent of America. The nurse was taking his temperature in the usual way, by placing the thermometer in his mouth, when the patient suddenly coughed and swallowed the instrument. The doctor in charge was immediately sent for, and on arrival had the patient suspended by the heels and shaken with great care, so as not to break the thermometer. All this, however, without the desired effect, and the patient is now quite well with the instrument inside him. A contemporary, in commenting on this interesting case, suggests that the patient's voracity has been under-estimated, and that in reality he swallowed the nurse as well as the thermometer.

### NOTES FROM THE SCHOOLS.

### Royal College of Surgeons of England.

The annual election of Fellows into the Council of the Royal College of Surgeons was held on Thursday in the library of the college. The vacancies were occasioned by the retirement of Sir William MacCormac, Mr. Sibley, and Mr. Sydney Jones, the last-named not being a candidate for re-election. The successful candidates were Sir William MacCormac, Mr. Thomas Richard Jessop (of Leeds), and Mr. Walter Rivington. Out of 1,153 Fellows, 436 voted. Mr. James Curling Bates, L.S.A.Lond., of Guy's Hospital, was, at the last meeting of the Council, admitted a member of the college.

### Middlesex Hospital.

Earl Spencer presided on Tuesday, June 30, in the lecture theatre of the Middlesex Hospital Medical School, at the distribution of prizes to successful students. There were also present: Lady Spencer, Lord Sandhurst (Chairman of the Weekly Board), the Lady Superintendent (Miss Thorrold), Mr. J. W. Hulke, F.R.S., and several of the medical staff. A report was read by the Dean, Mr. A. Pearce Gould, F.R.C.S., which stated that 129 students had joined the school, and that 360 students had attended the school and the Hospital of Medical Instruction during the past twelve months. The report having been adopted, Lord Spencer delivered a brief address, in which he referred to the great national service rendered by the English medical profession. One of the results of medical skill was the enormous decrease in the death-rate in this country. With regard to the hospitals and schools of the metropolis, he said that during the last two months he had sat on the Committee of the House of Lords, and listened to the interesting evidence given before that Commission. He was struck with the extensive work carried on by those institutions among the teeming poor and enormous populations of the metropolis. Although some of them, no doubt, were not conducted as they should be, many were conducted with marked ability. In the management of the London hospitals there had of late years been a great improvement. The following students obtained scholarships and prizes: Entrance scholarships of 100 guineas and 60 guineas—Mr. A. E. Walter and Mr. H. P. Noble respectively; Broderip scholarships, of the same amounts—Mr. Arnold Lawson and Mr. T. T. Cockill;

### THE STUDENT OF MEDICINE—(*continued*).

Governors' prize of 20 guineas—Mr. T. Carwardine; Hetley clinical prize of 25 guineas—Mr. T. Carwardine; Lyell gold medal for practical surgery—Mr. T. Carwardine; exhibition in anatomy, ten guineas—Mr. A. P. Coker. Prizes.—Medicine—Mr. W. H. Gibson and Mr. T. Carwardine, equal; surgery—Mr. H. W. Gibson; practical surgery—Mr. H. W. Gibson and Mr. T. Carwardine, equal; practical midwifery—Mr. T. E. Lloyd; anatomy—Mr. R. A. Young; physiology—Mr. R. A. Young; dissections—Mr. F. N. Cookson; chemistry—Mr. C. Robinson.

#### Charing Cross Hospital.

At the annual distribution of prizes to the successful students of this hospital, Lord Carrington took the chair and gave out the prizes. Mr. Boyd, the Dean, first read his report, in which it was stated that the Royal Colleges had agreed with the propositions put forward by the General Medical Council, and that the five years curriculum would soon become a matter of law. He then referred to the present difficulties of London medical students with respect to the M.D. degree. Many of the teachers of the London medical schools would gladly welcome some system for the founding of a central London teaching University, in which each of the hospitals with medical schools attached would be separate colleges of this University, with the result that both the education and the examination of the students would be conducted by their several teachers. According to the recommendations of the Royal Commission, two years were allowed to the University of London to endeavour so to alter its constitution as to combine within itself the functions of a teaching University and of an Imperial examining board. The endeavour failed most signally on May 12 last, when Convocation rejected the proposed charter of the Senate by a decisive vote. On May 26 the Privy Council announced that they would almost immediately proceed to the consideration of the petition of University and King's Colleges, London, for a charter as the Albert University of London. This haste took all by surprise; no delay could be obtained, and the case of the medical schools had to be prepared and lodged at the Privy Council office within a month. It was heard on Tuesday last week, and the chief demands were that each school should have collegiate rank in the University and should be represented by one member in the Council. The Royal colleges had petitioned to be allowed to form the medical faculty of the new University, and matters were thus complicated a good deal, but if their petition was granted the student would reap a great advantage, in that the need for two examinations on the same subject, and, of course, two fees, would be avoided. The whole case now lay with the Privy Council. It was to be hoped that no considerations would cause the Privy Council to constitute a faculty of medicine which would not be a practical success, for if properly worked the medical faculty would be by far the strongest in any University for London. Lord Carrington then distributed the prizes, which were awarded as follows: The Llewellyn scholarship, certificate, and £25, to Mr. D. C. Johnston; the Golding scholarship, certificate, and £15, to Mr. W. J. Robertson; the Governors' clinical gold medal to Mr. A. W. W. Hoffman; the Pereira prize to Mr. H. D. Senior; the Anatomy prize (senior) to Mr. W. J. Robertson; the Anatomy prize (junior) to Mr. D. P. Gabell; the Physiology prize (senior) to Mr. E. B. Jones; the Physiology prize (junior) to Mr. G. B. Clarke; the prize for Practical Physiology to Mr. W. T. White; the Chemistry prize to Mr. C. S. Reed; the Practical Chemistry prize to Mr. P. J. Probyn; the Osteology prize to Mr. S. H. Hayward; the Medicine prize to Mr. H. H. Phillips; the Practical Medicine prize to Mr. G. H. Hooper; the Surgery prize to Mr. S. L. J. Steggall; the Minor Surgery prize to Mr. R. W. S. Christmas; the Therapeutics prize to Mr. A. W. W. Hoffman; the Materia Medica prize to Mr. W. J. Robertson; the Midwifery prize to Mr. J. H. Day; the Forensic Medicine and Toxicology prize to Mr. A. W. W. Hoffman; the Pathology prize to Mr. Harold Gardner; and the Dental Surgery first prize to Mr. R. N. Gracey. Lord Carrington, in his speech, commented on the recent progress made by the Australian medical schools, and said that the members of the medical profession in that colony were the first to attempt to take in hand the difficult problem of English federation. Sir Joseph Fayrer afterwards proposed a cordial vote of thanks to Lord Carrington for presiding and for his interesting address.

### APPOINTMENTS.

At the West Norfolk and Lynn Hospital, H. Calthrop Allison, M.R.C.S., L.S.A., has been appointed senior surgeon; and George Chadwick, M.R.C.S., L.S.A., surgeon. At the Paris Branch of the Girls' Friendly Society, J. H. Barnard, M.D.Paris, M.R.C.S.,

### THE STUDENT OF MEDICINE—(*continued*).

has been appointed honorary physician. At the Croydon General Hospital, A. B. Carpenter, M.B.Oxon., L.R.C.P.Lond., has been appointed medical officer. At the Sheffield General Infirmary, Arthur Jackson, M.R.C.S., has been appointed honorary surgeon; and Simeon Snell, L.R.C.P., M.R.C.S., reappointed honorary ophthalmic surgeon. At the Shepperton Sanitary District, Edward Kingsford, F.R.C.S.Eng., has been reappointed medical officer. At the York County Hospital, J. B. Lendrum, M.B., C.M.Aberd., has been appointed assistant house surgeon. At Haileybury College, Horace Savory, M.A., M.B., B.C.Camb., M.R.C.S., L.R.C.P., has been appointed resident medical officer. For the Crystal Palace Gas Company, Frank Taylor, M.A., M.B.Camb., has been appointed medical officer.

### VACANCIES.

The following vacancies are announced this week, the amount of salary in each case being given after the name of the institution:

#### Resident Medical Officers.

Birmingham General Dispenaary, Resident Surgeon—Salary £150, and board, &c.

Bridgnorth and South Shropshire Infirmary, House Surgeon—Salary £80, with board, &c.

Cumberland Infirmary, Carlisle, Assistant House Surgeon—Salary £40, with board, &c.

Derbyshire General Infirmary, Resident Assistant House Surgeon—Salary £10 for first six months, £25 for second six months, with board, &c.

Kensington Dispensary, Resident Medical Officer—Salary £125, with board, &c.

Lancashire County Asylum, Rainhill, near Liverpool, Assistant Medical Officer to act as Pathologist—Salary £200, board, &c.

Miller Hospital and Royal Kent Dispensary, Greenwich, S.E., Junior Resident Medical Officer—Salary £30, with board, &c.

National Sanatorium for Consumption and Diseases of the Chest, Bournemouth, House Surgeon and Secretary—Salary £120, with board, &c.

Royal South Hants Infirmary, Southampton, Assistant to the House Surgeon—Board, &c.

St. Mark's Hospital for Fistula, &c., City Road, E.C., House Surgeon—Salary £50, with board, &c.

Tunbridge Wells General Hospital, House Surgeon and Secretary—Salary £100, with board, &c.

Wolverhampton Eye Infirmary, House Surgeon—Honorarium £25, with board, &c.

#### Non-Resident Medical Officers.

Bloomsbury Dispensary, W.C., Dispenser—Salary £100.

Glasgow Maternity Hospital, Out-door Physician.

Grosvenor Hospital for Women and Children, Vincent Square, S.W., Assistant Physician.

Irvinestown Union (Edenney Dispensary), Medical Officer—Salary £135, and fees.

St. Thomas's Hospital Medical School, Demonstrator of Physiology—Salary £100.

### PASS LISTS.

#### Society of Apothecaries of London.

The following gentlemen passed:—

Surgery.—F. J. Brown, Manchester, Owen's College; A. Paling, Middlesex Hospital; A. C. Fenn, and G. W. Wickham St. Bartholomew's Hospital; E. M. Rooke and C. P. Morgan, Guy's Hospital; E. J. T. Jones, St. Thomas's Hospital; J. H. Sproat, Birmingham, Queen's College; H. S. Ware, Cambridge and St. Thomas's Hospital; W. R. Nicol, Montreal.

Medicine, Forensic Medicine, and Midwifery.—H. B. Bates, Liverpool, University College; E. M. Dobinson, Guy's Hospital; G. T. B. Blick, St. Mary's Hospital; W. E. Reilly, University College; F. J. Brown, Manchester, Owens College; J. H, Sproat, Birmingham, Queen's College.

Medicine and Forensic Medicine.—E. Lambert, Leeds, Yorkshire College.

Medicine and Midwifery.—H. C. Poweil, Charing Cross Hospital; C. A. Ryde, London Hospital.

Forensic Medicne and Midwifery.—C. A. Lapthorn, Middlesex Hospital; C. A. Standring, King's College.

Medicine.—M. H. Knapp, St. Mary's Hospital; F. Spurr, Middlesex Hospital; C. S. Lewis, St. George's Hospital.

Midwifery.—C. R Harper, Middlesex Hospital.

Forensic Medicine.—T. C. Hughes, Westminster Hospital; L. Roberts, St. Mary's Hospital.

To the following gentlemen was granted the diploma of the Society entitling them to practise medicine, surgery, and midwifery: Messrs. Bates, Blick, Brown, Dobinson, Hughes, Lambeth, Lapthorn, Roberts, Rooke, Ryde, Sproat, and Standring.

#### University of Dublin.

At a meeting of the University Caput, held last week, the following degrees were conferred:—

Bachelor in Medicine.—Arthur Forbes McCutcheon.

Bachelor in Medicine, Surgery, and Obstetrics.—David Richard-

### THE STUDENT OF MEDICINE—(*continued*).

son Goodlatte Corrigan, John George Gibbon, William Robert Griffin, Hugh Hunter (B.Ch. stip. cond.), Joseph Singer Jameson, Richard Percy Jones, Owen Fenner Joynson, George Abraham Moore, Richard Albert Moynan, Frederic John Myles, David Stuart Sleith.

Doctor in Medicine.—Edward Arthur Crampton Baylor, James Craig, William Richard Dawson, Henry Hobart S. Dorman, Fitzgerald Isdell, Edward Parker Norman, W. J. Nickson (*in absentia*).

Doctor in Science (Honoris Causa).—James Emerson Reynolds, Daniel Cunningham, John Mallett Purser.

William Richard Dawson, M.D., of Portadown, has been awarded the Travelling prize of one hundred guineas.

### Royal College of Surgeons in Ireland.

The following gentleman, having passed the necessary examination, has been admitted a Fellow of the College: Havelock H. R. Charles, M.D., M.Ch. Queen's University, Surgeon I.M.S.

### Royal College of Physicians of Edinburgh.

The following candidates have been elected by the College since December 31st. 1890:—

Fellows.—J. W. Martin, 57, Ferry Road, Leith; G. Hunter, 33, Palmerston Place, Edinburgh; D. D. D. Turner, 6, George Square, Edinburgh.

Members.—A. H. Douglas, 6, West Maitland Street, Edinburgh; J. M. Balfour, Juniper Green; R. F. C. Leith, 129, Warrender Park Road, Edinburgh; G. M. Robertson, Royal Asylum, Edinburgh; R. A. Fleming, 36, Drumsheugh Gardens, Edinburgh; A. L. Gillespie, 12, Walker Street, Edinburgh.

Licentiates.—V. F. Allen, co. Cork; G. Astin, Burnley; Margaret Ida Balfour, Edinburgh; J. Banerjee, Bengal; V. E. Barrow; Madras; J. Barry, Ireland; A. H. Barstow, Harrogate; G. Billing, Manchester; H. D'A. Blumberg, Ventnor; S. J. Bolton, Belfast; T. B. Brook, Cambridgeshire; Edith Mary Brown, Whitehaven; W. Buck, Lancashire; W. F. Chevers, Cheltenham; C. J. V. Coller, Cape Colony; H. Collier; Sheffield; R. J. Collier, Belfast; R. P. Cooke, Ballyfarnon; G. S. Crawford, co. Antrim; P. R. Crofton, co. Roscommon; G. K. Crossthwaite, Canada; W. H. Cuthbert, Oswestry; T. J. Davies, Llanrwst; J. W. Davis, Freetown; A. Dennison, Leeds; D. Doherty, Donegal; A. S. Duke, Dublin; C. R. Dunhill, Madras; L. D. L. Ellis, Manchester; W. J. Evans, Limerick; J. C. Fenwick, Bishopwearmouth; J. A. Fink, Calcutta; J. Gilmour, co. Derry; P. J. Godfrey, co. Tipperary; M. B. Gorman, Cork; J. Grout, London; T. Hamilton, Tyrone; W. H. Harbison, Melbourne; Beatrice Mary Harrison, Brighton; E. Hartley, Lancashire; P. J. Hatton, Birkenhead; D. L. Heggie, Canada; J. Hodgson, Colne; T. E. Hughes, Rhyl; W. R. W. James, Bengalore; R. Johnson, York; S. Kirkpatrick, Sligo; J. Lane, co. Cork; T. Leahy-Lynch, Dublin; C. G. Lester, Sydney; J. W. Lewis, Cardiganshire; C. B. Lucas, Lanarkshire; J. M'Cullough, co. Down; J. G. Mackintosh, Edinburgh; R. Maclean, Ross-shire: A. A. Macleod, Greenock; C. N. Macquarie, Argyllshire; E. G. MacSweeney, Cork; P. G. 'Mahony, Maliapuram; F. R. Mallet, Lancashire; T. Marshall, South Shields; C. K. Martin, Dublin; S. Martyn, Mazagon; A. B. Masani, Bombay; R. Meggitt, Barton-on-Humber; W. Melville, Bo'ness; G. E. G. Metcalfe, Blackheath; R. W. Morrow, co. Down; E. R. Morton, Canada; J. B. Munro, Coatbridge; W. H. Nash, Dublin; F. Nuttall, Lancashire; J. Orr, co. Antrim; J. E. J. Pegg, Birmingham; Agatha Porter, Glasgow; J. T. T. Ramsay, Dundee; J. Reid, Argyllshire; C. Robson, Lincolnshire; W. B. Rotheros, Queenstown; C. C Salmon, Victoria; R. Scott, Kinrossshire; P. A. Shore, Walsall; J. B. Smith, Montrose; C. E. Southwell, Leeds; A. W. Spinks, Bradford; P. Stainsby, Saltaire; W. D. Sweeny, co. Mayo; H. E. Taaffe, Londonderry; F. P. D Thomas, Chester; W. Thyne, London; B. Tomkys, Bilston; P. Ukarji, Bombay; W. H. D. Vanes, Worcestershire; G. Vert, Haddington; J. S. Wheeler, Fleet; J. Whyte, Argyllshire; A. E. Woodcock, Cleckheaton.

### ATHLETICS.

#### Edinburgh.

United Hospitals Athletic Club v. Edinburgh University Athletic Club.—This contest was held at the Powderhall Grounds, Edinburgh, on Tuesday, June 30th, and ended in the victory of London by six points to four. The London team consisted of nine men only, the rest being absent. The results of the events were: 120 Yards Hurdle Race: 1, T. M. Donovan and H. W. G. Lander, Edin., dead heat; 3, W. Kent-Hughes, Lond. B. C. Green did not turn out. Time, 18 sec. One Mile Race: 1, D. S. Duncan, Edin.; 2, H. A. Munro, Lond. Time, 4 min. 33 3-5 sec. Duncan, the Scottish champion, won by a splendid sprint in the last 150 yards. 100 Yards Race: 1, B. C. Green, Lond.; 2, H. T. Bell, Lond. Time, 11 sec. Throwing the Hammer: 1, T. M. Donovan, Edin., 95 ft; 2, M. M'Innes, Edin., 85 ft. 10 in.; 3, M. Breton, Lond., 73 ft. 3 in. High Jump: 1, H. T. Bell, Lond., 5 ft. 7½ in.; G. M. Bucher, Edin., and P. Pierre, Lond., tied at 5 ft. 6½ in. Half-Mile Race: 1, W. Kent-Hughes, Lond.: 2, A. Hay, Lond.; 3, G. Hume, Edin. Time, 2 min. 7 1-5 sec. Putting the Weight: 1, M. M'Innes, Edin., 36 ft. 4 in.; 2, T. A. Chalmers, Edin., 33 ft. 1 in. 3, M. Breton, Lond., 32 ft. 10 in. Long Jump: 1, B. C. Green, Lond., 20 ft. 10½ in.; 2, H. T. Bell, Lond, 20 ft. 3½ in.; 3, G. A. Fothergill, Edin., 18 ft. 4 in.; 4, R. Williams, Edin., 18 ft. Quarter-Mile Race: 1, B. C. Green, Lond.; 2, J. G. Turner, Lond.; 3, T. M. Donovan, Edin.; 4, H. W. G. Lander, Edin. Time, 52 4-5 sec. This and the mile were the two best races. Green's victory was very loudly applauded. Three-Mile Race: 1, H. A. Munro, Lond.; 2, G. W. Pollard, Edin. Time, 15 min. 59 sec. The weather was very fine, but a strong south-west wind spoilt the times. The two teams were photographed before the races, and a dinner in honour of the United Hospitals team was afterwards held.

